# The impact of intravenous dodecafluoropentane on a murine model of acute lung injury

**DOI:** 10.1186/s40635-023-00518-2

**Published:** 2023-06-16

**Authors:** Jarrod M. Mosier, Saad Sammani, Carrie Kempf, Evan Unger, Joe G. N. Garcia

**Affiliations:** 1 Department of Emergency Medicine, University of Arizona College of Medicine, 1501 N. Campbell Ave., AHSL 4170D, P.O. Box 245057, Tucson, AZ 85724-5057 USA; 2 Department of Medicine, Division of Pulmonary, Allergy, Critical Care, and Sleep Medicine, University of Arizona College of Medicine, Tucson, AZ USA; 3 Department of Radiology, University of Arizona College of Medicine, Tucson, AZ USA

## Abstract

**Introduction:**

Intravenous oxygen therapeutics present an appealing option for improving arterial oxygenation in patients with acute hypoxemic respiratory failure, while limiting iatrogenic injury from conventional respiratory management.

**Methods:**

We used an established two-hit murine model of acute lung injury (ARDS/VILI) to evaluate the effect of intravenous dodecafluoropentane (DDFPe) on oxygen saturation and bronchoalveolar lavage cell counts and protein levels. Twenty hours after challenge with intratracheal lipopolysaccharide, mice were intubated and ventilated with high tidal volumes (4 h) to produce acute lung injury. DDFPe (0.6 mL/kg) or saline was administered by IV bolus injection at the initiation of mechanical ventilation and again at 2 h. Oxygen saturation was measured every 15 min. Bronchoalveolar lavage was performed at the conclusion of the experiment.

**Results:**

The two-hit ARDS/VILI model produced substantial inflammatory acute lung injury reflected by markedly increased bronchoalveolar lavage (BAL) cell counts compared to BAL cell counts in spontaneous breathing controls (5.29 ± 1.50 × 10^–6^ vs 0.74 ± 0.014 × 10^–6^ cells/mL) Similarly, BAL protein levels were markedly elevated in ARDS/VILI-challenged mice compared with spontaneous breathing controls (1109.27 ± 223.80 vs 129.6 ± 9.75 ng/mL). We fit a linear mixed effects model that showed a significant difference in oxygen saturation over time between DDFPe-treated mice and saline-treated mice, with separation starting after the 2-h injection. DDFPe-treated ARDS/VILI-challenged mice also exhibited significant reductions in BAL cell counts but not in BAL protein.

**Conclusion:**

DDFPe improves oxygen saturation in a murine model of ARDS/VILI injury with the potential for serving as an intravenous oxygen therapeutic.

## Introduction

Perfluorocarbons are low molecular density molecules that are uniquely both hydrophobic and lipophobic and can dissolve large volumes of gas [1]. When emulsified with surfactant, the perfluorocarbon emulsion provides an opportunity for intravenous oxygen transport. This could be potentially useful in oxygenation threatening diseases from embolism (e.g., stroke, heart attack), hemorrhage, or acute lung injury. Acute lung injury, especially the most severe form–acute respiratory distress syndrome, presents significant clinical challenges during noninvasive respiratory support, preoxygenation for tracheal intubation, and during mechanical ventilation largely because of ventilation:perfusion mismatch and intrapulmonary shunt.

Intravenous perflurocarbons are a potential novel solution for these challenges posed by acute lung injury. While intratracheal perfluorocarbons have shown harm in clinical trials for liquid ventilation, [2] *intravenous* perfluorocarbons have potential as a small particle oxygen therapeutic. Dodecafluoropentane emulsion (DDFPe) [NanO_2_™, NuvOx Pharma (Tucson, AZ)] is one such promising perfluorocarbon. DDFPe_,_ is 2% weight/volume dodecafluoropentane emulsion, with a lower boiling point, higher oxygen carrying capacity at lower doses, and improved safety profile compared to previous perfluorocarbons, [3, 4] which make it an ideal candidate for intravenous O_2_ delivery [5]. In this study, we used an established preclinical model of acute lung injury to evaluate the potential of DDFPe to improve oxygen saturation in conditions of low ventilation:perfusion and high intrapulmonary shunt.

## Methods

### Murine model

We used an established two-hit mouse model of acute lung injury for these experiments [6, 7] to model acute respiratory distress syndrome with hypoxemia to evaluate the effect of bolus doses of DDFPe on oxygen saturation, and to avoid using an unestablished model of apnea. C57BL/J6 male mice (*n* = 14, 8–10 weeks) were used for the experiments. Twelve mice were anesthetized with intraperitoneal ketamine (100 mg/kg) and xylazine (5 mg/kg), and then intubated with a 20-gauge intravenous catheter. Lipopolysaccharide (0.5 mg/kg) was then given by intratracheal injection, mice were extubated and allowed to recover for 20 h. After the recovery period, the mice were re-sedated with ketamine and xylazine by intraperitoneal injection, with additional doses and bupremorphone (0.3 mg/kg) given as needed for pain and to ensure deep sedation during the experiment, reintubated and placed on a mechanical ventilator (Harvard Apparatus, Boston, MA).

At the onset of mechanical ventilation, mice were randomly assigned to injection with either DDFPe (0.6 mL/kg) or an equivalent volume of saline (*n* = 6/group) into the surgically exposed right jugular vein. A second bolus was given at 120 min. Mice were ventilated to induce ventilator-induced lung injury (VILI) throughout the experiment by using a tidal volume of 30 mL/kg, respiratory rate of 75 breaths/min, and ambient FiO_2_ without positive end-expiratory pressure for four hours. Handlers were not blinded to treatment assignment.

Oxygen saturation was continuously monitored using the MouseStat^®^ monitor (Kent Scientific Corporation, Torrington, CT) on the pad of a hind leg paw and measurements were recorded as an average over 30 s every 15 min. We fit a linear mixed effects model to the oxygen saturation data with a random intercept per mouse, and conducted linear hypothesis tests of group differences at each time point using Kenward–Roger adjustments to the denominator degrees of freedom and standard errors.

At the termination of each experiment (after 4 h of mechanical ventilation), bronchoalveolar lavage (BAL) fluid was collected by instilling 1 mL of HBSS (Invitrogen, Grand Island, NY) through the tracheal catheter, followed by slow recovery of the fluid. The remaining two mice did not undergo any study interventions (i.e., anesthesia and intubation for LPS injection, or mechanical ventilation), except anesthesia to facilitate bronchoalveolar lavage. Cells were recovered from the resulting bronchoalveolar lavage fluid by centrifugation (500 g, 20 min, 4 °C) and counted using an automated cell counter (TC20; Bio-Rad, Hercules, CA). BAL indices are reported as mean ± standard deviation and compared using a student’s *t* test. All animal care procedures and experiments were approved by the University of Arizona Animal Care and Use Committee (Approval #13-490).

## Results

**Fig. 1:**
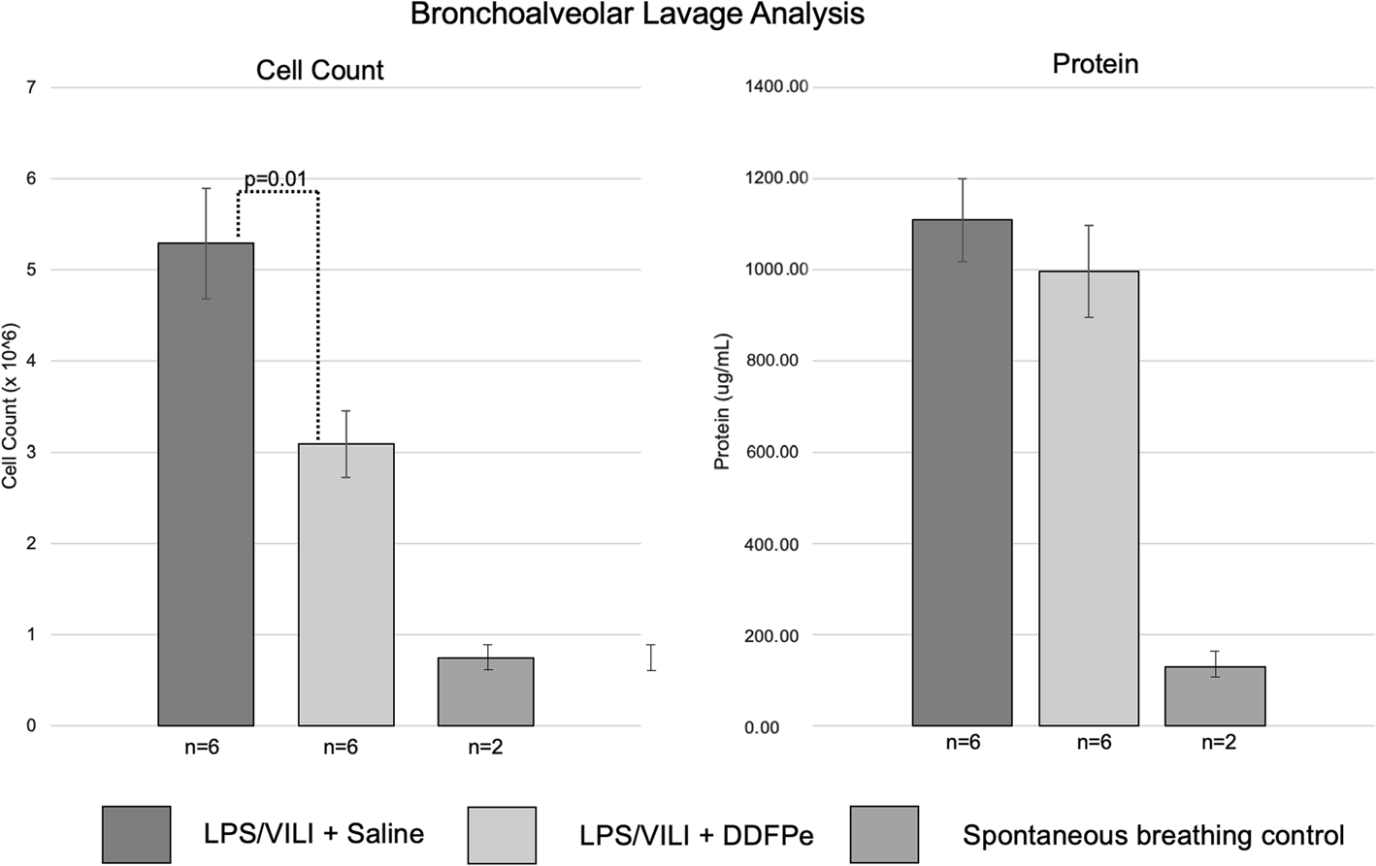
Bronchoalveolar lavage analysis at the end of the experiment demonstrated an inflammatory acute lung injury with the two-hit model for both saline-treated and DDFPe-treated mice (*n* = 12) compared to spontaneously breathing mice that were not exposed to LPS injection or mechanical ventilation (*n* = 2). There was a statistically significant decrease in cell count (*p* = 0.01) with DDFPe-treated mice compared to saline-treated mice (dashed line, left panel), but no difference in protein concentration (*p* = 0.33) (right panel). Error bars represent standard deviation

The two-hit model produced the predicted inflammatory acute lung injury with an increased bronchoalveolar lavage cell count in the DDFPe (3.08 ± 0.88 cells/mL, *n* = 6) and saline (5.29 ± 1.50 cells/mL, *n* = 6) groups compared to spontaneous breathing unexposed control mice [0.74 ± 0.014 × 10^–6^ cells/mL, (*n* = 2)]. Mice exposed to ARDS/VILI also exhibited an increase in BAL protein compared to spontaneous breathing unexposed control mice [DDFPe: 996.89 ± 246.8, saline: 1109.27 ± 223.80 vs 129.6 ± 9.75) ng/mL]. Examination of BAL protein values showed no difference in protein content between the saline-treated and the DDFPe-treated groups but a significant reduction in BAL cell count in mice receiving DDFPe (Fig. 1).

**Fig. 2:**
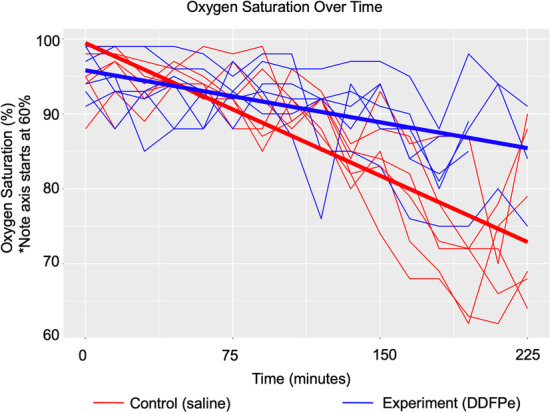
Oxygen saturations over time for mice treated with saline (red) and mice treated with DDFPe (blue), *n* = 6 for each group. Raw data are presented (thin lines) along with overlayed model estimates (thick). Time zero is the initiation of mechanical ventilation with injurious tidal volumes, 20 h after intratracheal lipopolysaccharide injection. DDFPe or saline was given at 0 and 120 min

Oxygen saturation measurements were variable between mice at each measurement (Fig. 2). The mean oxygen saturation at the initiation of mechanical ventilation was 95–96% for both the saline-control group and the DDFPe-treated group, but steadily declined to a mean of 91% for saline controls and 89% for DDFPe-treated mice over the first 2 h. After 4 h of ARDS/VILI exposure, the mean oxygen in the saline-treated group fell further to 76% with a mean 4 h difference of − 19%. In contrast, mean oxygen saturation levels in the DDFPe treated group exhibited a mean 4 h difference of -10%. In the linear mixed effects model, there was an overall significant difference between the DDFPe-treated and saline-treated mice (*p* < 0.001), and the linear estimates show separation between the groups starting at the 2-h dose (Table 1).

**Table 1:** Linear hypothesis tests of differences between DDFPe and saline treated mice (*n* = 6/group) at each time point using Kenward–Roger adjustments to the denominator degrees of freedom and standard errors

Time point	Estimate difference	t statistic	df	*p* value	95% CI
Baseline**	− 3.63	− 1.75	22.18	0.09	− 7.93, 0.67
15 min	− 2.56	− 1.29	18.72	0.21	− 6.71–1.60
30 min	− 1.48	− 0.77	15.96	0.44	− 5.52–2.56
45 min	− 0.40	− 0.21	13.81	0.82	− 4.34–3.54
60 min	0.67	0.37	12.21	0.71	− 3.20–4.54
75 min	1.75	1.00	11.07	0.33	− 2.07–5.57
90 min	2.82	1.65	10.35	0.12	− 0.9–6.62
105 min	3.90	2.30	10.02	0.04	0.13–7.67
120 min**	4.98	2.94	10.07	0.01	1.21–8.76
135 min	6.05	3.54	10.48	0.005	2.26–9.85
150 min	7.14	4.09	11.29	0.002	3.31–10.97
165 min	8.21	4.58	12.52	< 0.001	4.33–12.10
180 min	9.29	5.01	14.24	< 0.001	5.33–13.25
195 min	10.37	5.39	16.52	< 0.001	6.30–14.43
210 min	11.44	5.71	19.42	< 0.001	7.25–15.63
225 min	12.52	5.98	23.04	< 0.001	8.19–16.85

**DDFPe or saline injection

The change in oxygen saturation after the dose of DDFPe or saline at 2 h under hypoxemic conditions was interesting. The difference between the mean O_2_ saturation immediately prior to injection and the mean O_2_ saturation after injection in the control saline group was − 7.5% (91% and 83%, respectively), whereas mean O_2_ saturation in the NanO_2_ treated group rose from 89% to 91%, mean difference + 2.5%, net difference 10% [95% CI: 2.7,17.3], *p* = 0.01).

## Discussion

These results show that in a ‘two-hit’ model of acute lung injury, intravenously administered DDFPe improved oxygen saturation. The mice had similarly declining oxygen saturation trajectories until 2 hours when the second injection of DDFPe while hypoxemic rapidly increased oxygen saturation and changed the trajectory for the remainder of the experiment. These results indicate that DDFPe is a viable candidate for an intravenous oxygen therapeutic. Although the mechanism of action is not completely understood, DDFPe appears to improve arterial oxygen content despite disruptions in ventilation:perfusion mismatch induced by both endothelial (lipopolysaccharide) and epithelial injury (volutrauma).

It is interesting that a reduction in bronchoalveolar lavage cell count, but not total protein was observed in DDFPe-treated mice compared to controls. One hypothesis is that DDFPe may reduce oxidative stress leading to reduced cellular infiltration but not affecting capillary leak. This is aligned with a study by Hou and colleagues that showed perfluorooctyl bromide (Oxygent) reduced pulmonary edema and inflammatory cellular infiltration, and improved PaO2 when given prophylactically in an LPS induced acute lung injury model [8]. In our study, LPS induced injury had progressed for 20 h before the onset of the “second hit” of injurious mechanical ventilation. However, this hypothesized mechanism for DDFPe requires further exploration as a possible alternative explanation is less severe injury in the DDFPe-treated mice.

DDFPe has demonstrated efficacy in preclinical models in reducing cerebral damage from acute ischemic stroke, [9–12] and decreasing myocardial damage in acute myocardial infarction [13]. DDFPe was also shown to rapidly improve PaO_2_ in a porcine model of intrapulmonary shunt induced by bead instillation into the bronchial tree [14], as well as improve tissue hypoxia in preclinical hemorrhage, stroke, and acute chest syndrome models [11, 15, 16]. Our results add to the existing preclinical studies demonstrating the potential for DDFPe to restore systemic oxygenation and attenuate hypoxic tissue injury. In addition, DDFPe can be administered repeatedly to restore oxygenation [14], which increases the therapeutic utility for treating acute hypoxemic respiratory failure across the spectrum of disease.

The improvement in oxygen saturation seen in this study is a potentially clinically translatable option for overcoming the challenges imposed by ventilation:perfusion mismatch and shunt in patients with acute hypoxemic respiratory failure. Tracheal intubation in particular is dangerous in this patient population, as it carries significant risk of oxygen desaturation leading to peri-intubation cardiac arrest [17, 18]. This risk is attenuated by preoxygenation, but the most severe cases with high intrapulmonary shunt are often refractory to preoxygenation and drastically limit, or eliminate, the possibility of a safe apnea time [19–21]. In these cases, pulmonary blood flow is not resaturated by the high oxygen content of the functional residual capacity and patients rapidly desaturate [19]. A single dose of DDFPe during the preoxygenation period could potentially improve oxygen saturation and reduce the risk of critical desaturation during intubation. After the 2-h dose of DDFPe, when the mice were hypoxemic, the mean difference in oxygen saturation was 10% higher in DDFPe-treated mice compared to saline-controls. Future experiments under apnea while hypoxemic are warranted to further evaluate the potential efficacy for this indication.

Research is needed using various dosing strategies to evaluate the potential role of DDFPe in acute hypoxemic respiratory failure patients. Studies are also needed on the mechanism of action and safety in humans. Perfluorocarbons have the potential to create microemboli in the pulmonary circulation [1], which could consequentially worsen dead space in a patient with ARDS, and tert-butylcyclohexane was shown to create thrombocytopenia in an animal model of LPS induced inflammation [22]. However, repeated doses of DDFPe were well-tolerated in human trials for stroke [16]. Finally, a more complex analysis model to allow an evaluation of non-linear effects of the data may be further revealing and future experiments should include this approach. However, there are not enough degrees of freedom in our data to allow such a model, and the distribution of our residuals are normally distributed and do not show obvious heteroscedasticity that would indicate such a model is necessary.

These results indicate that DDFPe is an appealing candidate for improving arterial oxygenation in the presence of ventilation:perfusion mismatch and intrapulmonary shunt.

## Data Availability

Data are available with appropriate ethics review and data use agreement.

## Abbreviations

DDFPe: Dodecafluoropentane emulsion
VILI: Ventilator-induced lung injury
BAL: Bronchoalveolar lavage
ARDS: Acute Respiratory Distress Syndrome
